# Endocannabinoid-mediated regulation of depression in the ovBNST

**DOI:** 10.3389/fnins.2025.1629351

**Published:** 2025-07-25

**Authors:** Riming Zhu, Jie Li, Xia Zhang, Bin Zhang

**Affiliations:** ^1^Qingdao Medical College of Qingdao University, The Affiliated Hospital of Qingdao University, Qingdao University, Qingdao, China; ^2^Department of Neurology, West China Hospital of Sichuan University, Chengdu, China

**Keywords:** ovBNST, endocannabinoid system, CB1R, chronic stress, antidepressant effect

## Abstract

**Introduction:**

The bed nucleus of stria terminalis (BNST) acts as a crucial hub for assessing vigilant threats, with the oval subnucleus (ovBNST) being enriched in endocannabinoid ligands and receptors. The endocannabinoid system (ECS) is well recognized for its role in stress responses. However, the molecular and circuitry mechanisms through which the ovBNST ECS mediates chronic stress induced depressive phenotypes remain unclear.

**Methods and results:**

The chronic unpredictable mild stress (CUMS) was optimized to model the depression-like behaviors and body weight loss in mice. By utilizing the endocannabinoid sensor, an increased release of endocannabinoid in the ovBNST was probed in response to acute stress. Local blockage of ovBNST cannabinoid type 1 receptor (CB1R) with NESS0327 induced both anhedonia and despair depressive phenotypes in naïve mice. In contrast, intra-ovBNST infusion of either CB1R agonist or cannabinoid hydrolase inhibitor JZL-184 ameliorated despair-like behaviors while merely changed anhedonia in CUMS mice. By combining viral tracing with RNAscope and western blotting, the reduction in CB1R transcriptional and translational level was found to be associated with the CUMS induced depressive disorders. This reduction may be attributed to the changes in ovBNST located presynaptic CB1R that originates from the medial prefrontal cortex (mPFC).

**Discussion:**

Overall, these results suggest that chronic stress may restructure the ovBNST ECS to result in depressive phenotypes. This study may extend the comprehension of ECS in the ovBNST, specifically its role in modulating the pathogenesis of depressive disorders induced by chronic stress.

## Graphical Abstract

The CUMS is associated with decreased levels of cnr1 mRNA in the mPFC and a corresponding reduction in the CB1R protein in the ovBNST, leading to the endocannabinoid-mediated depressive phenotypes. Intra-ovBNST administration of the CB1R antagonist NESS0327 induces depression-like behaviors in naïve mice, whereas intra-ovBNST infusion of the MAGL inhibitor JZL-184 ameliorates depression-like behaviors in CUMS mice. 
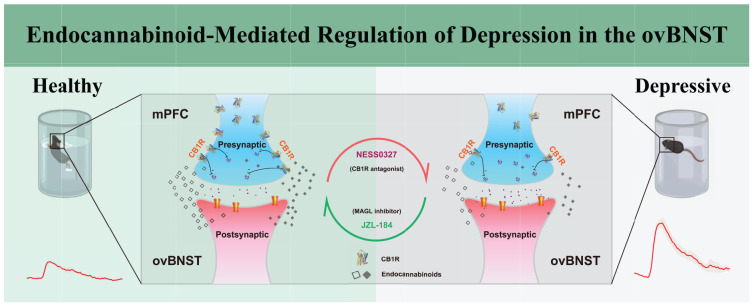

## 1 Introduction

Depressive disorder affects approximately 280 million people in the world (World Health Organization, 2021; GBD 2019 Mental Disorders Collaborators, 2022; World Health Organization, 2017). Despite the high morbidity and substantial burden, the mechanisms underlying the pathogenesis of depressive disorder remain elusive.

Numerous studies have implicated that multiple brain areas contribute to the development of depressive disorder (Nestler et al., 2002). For instance, the nucleus accumbens (NAc) related reward circuitry alleviates anhedonia in depressed mice (Heshmati and Russo, 2015) while the lateral habenula (LHb) related anti-reward circuitry exacerbates anhedonia in naïve mice (Yang et al., 2018). The BNST comprises distinct neuronal subpopulations characterized by various neuromodulators and receptors,

facilitating the monitoring of emotional valence (Lebow and Chen, 2016). The ovBNST acts as a node of threat assessment (Kim et al., 2013; Avery et al., 2016; Han et al., 2012; Zhang et al., 2021; Calhoon and Tye, 2015). Previous research has demonstrated that the kappa opioid receptor agonist U-50488, which produces depressive-like effects, elevates cFos expression in the ovBNST (Russell et al., 2014).

Although previous research indicates that the corticotropin-releasing hormone and protein kinase C-d-positive neurons in the ovBNST function oppositely in stress responses (Wang et al., 2020; Williford et al., 2023), the ovBNST exhibits a high degree of complexity in its neuromodulator composition that need further investigation. In addition to heterogenous neuropeptides, the bioactive lipid system, particularly the endocannabinoid system (ECS), has been identified within the ovBNST and is implicated in the modulating stress responses (Crestani et al., 2013; Puente et al., 2011; Tsou et al., 1998). The ECS comprises ligands, receptors, and metabolic enzymes that are responsible for endocannabinoid synthesis and degradation, representing an important substrate for the control of stress resilience, pain perception, and cognitive function through the activation of presynaptic or astroglial CB1R by two endocannabinoid ligands, anandamide (AEA) and 2-arachidonoylglycerol (2-AG) (Iversen, 2003; Zou and Kumar, 2018; Mechoulam and Parker, 2013; Han et al., 2012; Huang et al., 2016). AEA is primarily synthesized postsynaptically via an Nacyl-phosphatidylethanolamine (NAPE) pathway and is cleaved in both presynaptic and postsynaptic compartments by fatty acid amide hydrolase (FAAH) (Maccarrone et al., 2015; Maccarrone, 2017; Kilaru and Chapman, 2020). On the other hand, 2-AG is produced by postsynaptic diacylglycerol lipases (DAGL) and degraded by presynaptic monoacylglycerol lipase, which can be selectively inhibited by the compound JZL-184 (Maccarrone et al., 2015; Kilaru and Chapman, 2020; deRoon-Cassini et al., 2020; Maccarrone, 2020). Deficiency of CB1R induces depressive-like behaviors in mice (Beyer et al., 2010; Valverde and Torrens, 2012; Shen et al., 2019). On the contrary, elevating the AEA level produces antidepressant effects (Wang and Zhang, 2017). The anti-depressant actions of JZL-184, an inhibitor of monoacylglycerol lipase (MAGL) that degrades 2-AG, were observed in both naïve mice and chronic stress-treated mice (Wang and Zhang, 2017). Notably, the CB1R expressed in the BNST has been confirmed to encode emotional valence and shift phasic fear to sustained anxious apprehension (Massi et al., 2008; Glangetas and Georges, 2016; Lange et al., 2017; Gomes-de-Souza et al., 2021). However, the precise mechanism by which the ovBNST ECS regulates depressive disorder remains largely unexplored. The high enrichment of CB1Rs in the ovBNST led to the hypothesis that the ovBNST ECS exerted a regulatory control over depressive disorder.

To verify the hypothesis, the CUMS paradigm was optimized to establish a stable mouse model of depression. Subsequently, fiber photometry was utilized to examine dynamic changes of the neuronal activity and endocannabinoid release in the ovBNST during acute stress. Finally, the role of the ECS in the mPFC-ovBNST circuit in regulating depression-like behaviors was elucidated through behavioral tests, neuropharmacological manipulation and *in situ* hybridization.

## 2 Materials and methods

### 2.1 Animals

Male C57BL/6J mice, aged 8 weeks, were utilized in the experiments. The mice were procured from Beijing Vital River Laboratory Animal Technology Co., Ltd., and group-housed (4–5 mice per cage) in an exhaust ventilated closed-system (Fengshi, Suzhou) before the experiment. The cages were maintained in a temperature-controlled environment (24 ± 2°C) with a relative humidity of 60% ± 10% and a 12 h light/dark cycle (lights on from 7:00 AM to 7:00 PM). The cages were cleaned twice a week to ensure a hygienic living environment for the mice. Except for brief periods of food deprivation and water deprivation during the stress modeling, Laboratory chow (purchased from Jiangsu Xietong Pharmaceutical Bio-Engineering Co., Ltd.) and sterile water were provided *ad libitum* to ensure the nutritional needs and hydration of the mice throughout the study. All animal procedures were approved by the Qingdao University Animal Care Committee (QDU-AEC-2024364).

### 2.2 CUMS model

The CUMS protocol was implemented, with slight modifications to the original method by Willner et al. (Willner et al., 1987; Willner et al., 1992). This protocol incorporated both short-term and long-term stressors, over a total duration of 4 weeks (Table 1). Short-term stressors comprised forced swimming for 6 min, tail suspension for 15 min, physical restraint for 2 to 6 h, light flash for 12 h, inverted light/dark cycle for 12 h, and tail pinch for 15 min. Long-term stressors included 24 h wet bedding, 24 h food or water deprivation, 24 h soiled bedding, 24 h sleep deprivation (using Rotating-Bar Sleep Deprivation Apparatus, SA108, SansBio), and 24 h tilted cage. Short-term stressors were randomly applied to single-housed mice during the day or night, in conjunction with long-term stressors, over a total duration of 4 weeks. Single-housed control mice were remained undisturbed. All efforts were made to minimize stress and ensure the well-being of the animals throughout the study. Following the completion of the CUMS modeling, mice were allowed to recover for 1 day before subsequent behavioral tests. Continuous weighing was conducted on the morning of Day 0, 7, 14, 21, and 28 before applying water deprivation or food deprivation to mitigate their influence on body weight changes.

**TABLE 1:** The CUMS protocol.

Time	Monday	Tuesday	Wednesday	Thursday	Friday	Saturday	Sunday
1 Week	C + 2 + D	A + 6 + D	B + 4 + D	C + 5 + D	F + 3 + D	D + 2 + D	E + 6 + D
2 Week	C + 2 + D	B + 6 + D	E + 4 + D	F + 5 + D	A + 6 + D	E + 1 + D	B + 4 + D
3 Week	A + 1 + D	E + 5 + D	B + 5 + D	C + 1 + D	B + 3 + D	A + 4 + D	E + 1 + D
4 Week	E + 3 + D	C + 2 + D	A + 6 + D	B + 4 + D	F + 3 + D	A + 1 + D	C + 3 + D

**Short-term stressors:** A: forced swimming 6 min, B: tail pinch 15 min, C: tail suspension 15 min, D: physical restraint 2−6 h, E: inverted light/dark cycle 12 h, F: light flash 12 h. **Long-term stressors:** 1: wet bedding 24 h, 2: water deprivation 24 h, 3: soiled bedding 24 h, 4: sleep deprivation 24 h, 5: food deprivation 24 h, 6: tilted cage 24 h.

### 2.3 Virus

The recombinant adeno-associated viruses AAV2/9-hSyn-jGCaMP7s-WPRE-pA (Taitool, Cat# S0589-9-H50, 4.90 × 10^12^ vg/mL), AAV2/2-RetroPlus-hSyn-Cre-EGFP-WPRE-pA (Taitool, Cat# S0230-2RP-H50, 5.01 × 10^12^ vg/mL), AAV2/9-hSyn-eCB2.0 (BrainCase, Cat# BC-0286, 5.45 × 10^12^ vg/mL) (Dong et al., 2022) were purchased from Taitool (Shanghai) and BrainCase (Shenzhen).

### 2.4 Drug treatment

The CB1R antagonist NESS0327 (NESS, Cat# HY-117139), the CB1R antagonist AM251 (Cat# HY-15443), and the MAGL inhibitor JZL-184 (Cat# HY-15249) were purchased from MedChem Express. The CB1R agonist WIN55212-2 (WIN, Cat# W102) was obtained from Sigma-Aldrich. The neutral antagonist NESS (0.03 μg/100 nL/site), WIN (0.5 μg/100 nL/site), and the antagonist/inverse agonist AM251 (0.25 μg/100 nL/site) were dissolved in a vehicle mixture (DMSO, Tween-80, and artificial cerebrospinal fluid (ACSF) in a 1:1:98 volume ratio) (Pertwee, 2001). JZL-184 (0.5 μg/100 nL/site) was dissolved in another vehicle mixture (DMSO, Tween-80, and ACSF in a 5:1:94 volume ratio) (Pertwee, 1997; Pan et al., 2011). All drugs were locally administered via an integrated bilateral cannula (250 μm in inner diameter, KedouBC, Suzhou) 30 min before the behavioral test, with the injection needle retained for 2 min to facilitate absorption.

### 2.5 Stereotaxic surgery

Mice were anesthetized with 1% sodium pentobarbital and carefully immobilized in a stereotaxic frame (RWD Life Science Co., Ltd., China). A midline incision was made to expose the skull. The vertical height difference between the bregma and lambda reference points was within 0.03 mm.

Viral Injection: A 10 μL microsyringe connected to a micro-injection pump (Legato 130, KD Scientific Inc), was used to inject the virus at a rate of 10–20 nL/min into the mPFC (AP: + 1.90 mm, ML: −0.30 mm, DV: −2.20 mm) or ovBNST (AP: + 0.26 mm, ML: −1.00 mm, DV: −3.95 mm).

Optic Fiber or Cannula Implantation: A sterilized optic fiber or cannula was accurately lowered above the ovBNST (AP: + 0.26 mm, ML: ± 1.00 mm, DV: −3.85 mm).

The wound was managed post-surgery with lidocaine/chloramphenicol ointment to deliver pain relief and mitigate the risk of infection. Mice were returned to the animal facility for housing after 24-h monitoring of vital signs.

### 2.6 Behavioral tests

#### 2.6.1 Sucrose preference test (SPT)

Mice were given 24 h access to two water bottles in single-cage housing for training. Every 12 h, the bottles were switched and weighed. Mice showing a side preference exceeding 75% were excluded. The training was repeated with a 1% sucrose solution for another 24 h, followed by 24 h water deprivation. A 2 h SPT was then conducted, with bottle switching after 1 h. Sucrose preference was calculated by dividing the total consumption of sucrose solution by the total consumption of both water and sucrose solution.

#### 2.6.2 Open field test (OFT)

Mice were placed individually in a white, square arena (45 × 45 × 45 cm). Behaviors were recorded for a period of 10 min and analyzed with EthoVision XT 17 software (Noldus, Netherlands) to automatedly quantify the total distance traveled and the duration of time spent in the center (22.5 × 22.5 cm).

#### 2.6.3 Forced swimming test (FST)

Each mouse was gently placed in a transparent cylindrical tank (12 cm diameter, 30 cm height) containing warm water at 23–25°C for a period of 6 min. The main measurement was the duration of immobility observed during the final 4 min. Animal behaviors were captured using the Bandicam Screen Recorder software (version 7.1.2158, Bandicam Company, Republic of Korea). The immobile duration during the last 4-min test was counted offline by an observer blinded to animal treatment. Immobility was defined as a state in which animals remained floating or motionless, exhibiting only movements necessary for maintaining balance in the water.

#### 2.6.4 Elevated plus maze (EPM) test

The EPM consists of open arms and closed arms, elevated 50 cm above the floor. Animal behaviors were recorded over a 5 min period. The number of entries into the open arms, time spent in the open arms, and total movement distance were measured.

The time spent in the open arms and total distance traveled were analyzed with EthoVision XT 17 software.

#### 2.6.5 Tail suspension test (TST)

Mice were suspended by the tail with adhesive tape placed one-quarter of the length from the tip, ensuring the head was 30 cm above the ground. The immobility time during the last 4 min of the 6 min test was assessed. To continuously assess the TST performance, tests were conducted on the morning of Day 0, 7, 14, 21, and 28 following weighing throughout the 4-week CUMS modeling period. Behaviors were recorded using Bandicam Screen Recorder software. The immobile duration during the last 4-min test was counted offline by an observer blinded to animal treatment. Immobility was defined as a state in which animals ceased attempting to escape or move around.

### 2.7 *In vivo* fiber photometry

Two weeks after AAV2/9-hSyn-jGCaMP7s expression in the ovBNST or AAV2/9-hSyn-eCB2.0 expression in the mPFC, an optical fiber (O.D. = 400 μm, NA = 0.37, Inper) was implanted above the ovBNST. A three-color multi-channel fiber photometry system (Nanjing ThinkerTech, China) was used to record jGCaMP7s and eCB2.0 signals excited at 470 nm (20–30 μW) and the control signal excited at 410 nm (10 μW) at 100 Hz. Recorded data were visualized and analyzed using Matlab. The jGCaMP7s and eCB2.0 signals were compensated for and corrected by referencing the 410 nm control channel to minimize the inference of bleaching and noise prior to further analysis. Fluorescence signals were segmented into distinct time intervals, each defined by specific environmental conditions. The change in fluorescence intensity (ΔF/F) was determined by the formula (F-F_0_)/F_0_-F_*offset*_, where F denotes the current fluorescence intensity and F_0_ is the baseline fluorescence intensity, chosen based on the experimental context. Data were presented as peri-event plots, reflecting the average outcomes across multiple experimental trials. To represent the cumulative fluorescence intensity during a brief period of stress, area under the curve (AUC) was calculated as the integral of the ΔF/F signal over a 6-s time window.

### 2.8 Immunohistochemistry

Mice were deeply anesthetized with an overdose of sodium pentobarbital (60 mg/kg, intraperitoneal injection). Once a lack of response to toe pinch was confirmed, the mice were transcardially perfused with 0.9% saline and 4% paraformaldehyde. The post-fixed and dehydrated brain was sectioned into 30 μm slices using a cryostat microtome (CM1860, Leica). The slices received antigen retrieval, permeabilization, and blocking, sequentially. Primary antibody (Rabbit Anti-cFos, Synaptic Systems, 1:4000; Goat Anti-GFP, Rockland, 1:1000) incubation was performed overnight at 4°C followed by secondary antibody incubation. The sections were imaged using a laser scanning confocal microscope (FV3000, Olympus).

### 2.9 RNAscope *in situ* hybridization

The RNAscope Fluorescent Multiplex Assay kit (Advanced Cell Diagnostics (ACD), Cat# 323100) was conducted in accordance with the user guide. Briefly, block the rehydrated slides with 200 μL RNAscope^®^ Hydrogen Peroxide solution (ACD, Cat# 322381) at room temperature (RT) for 10 min, followed by target retrieval with RNAscope^®^ Target Retrieval Reagent (ACD, Cat# 322000, diluted to 1 × before use) using a 95°C water bath for 5 min. After cooling to RT, slides underwent before RNAscope^®^ Protease III digestion (ACD, Cat# 322381) at 40°C for 20 min to enhance probe penetration. Pretreated slides were incubated with *cnr1* probe (RNAscope^®^ Probe-Mm-*cnr1*-C3, 1:50, ACD, Cat# 420721) at 40°C for 2 h. Subsequently, AMP1, AMP2, and AMP3 reagents were added, followed by detection with HRP-C3 and TSA Vivid 570 (RNAscope^®^ Multiplex Fluorescent Detection Regents v2, ACD, Cat# 323110). Images were obtained utilizing the Olympus FV3000 confocal microscope. A minimum of five mice, with one image taken per mouse, were included for subsequent analysis in ImageJ (version 1.54j, National Institutes of Health, United States).

### 2.10 Western blotting

Brain tissues were homogenized in RIPA lysis buffer (Beyotime, Cat# P0013B) supplemented with protease and phosphatase inhibitors (cOmplete™ Protease Inhibitor Cocktail, Merck, Cat# 04693116001), followed by centrifugation at 12,000 × g for 30 min at 4°C to collect the supernatants. Protein concentrations were determined using a BCA assay kit (Beyotime, Cat# P0012), followed by denaturation at 100°C for 5 min. A total of 25 μg protein per sample was loaded into each lane and was separated on a 10% SDS–PAGE gel, which was subsequently transferred onto PVDF membranes (Beyotime, Cat# FFP32) for western blotting analysis. PVDF membranes were blocked with skimmed milk, and incubated overnight at 4°C with primary antibodies (anti-CB1R, Abcam, Cat# ab259323, 1:1,000; anti-GAPDH, Beyotime, Cat# AF1186, 1:1,000). After washing, PVDF membranes were incubated with an HRP-conjugated secondary antibody (Beyotime, Cat# A0208, 1:1,000) for 2 h at RT. Signal detection was performed using a high-sensitivity ECL detection solution (Beyotime, Cat# P0018AS). All bands were visualized using the Fusion FX imaging system (VILBER, France) and quantified using densitometric analysis in ImageJ.

### 2.11 Data analysis

Experimental data are expressed as the mean ± SEM. Statistical analyses were conducted with Prism software (version 9.4, GraphPad Company, San Diego, CA, United States). Data comparisons employed either one-way ANOVA or Student’s *t*-test, unpaired or paired as appropriate. *Post hoc* analysis following one-way ANOVA was performed using the *Tukey-Kramer* test. Statistical significance was set at *P < 0.05, **P < 0.01, ***P < 0.001, ****P < 0.0001.

## 3 Results

### 3.1 Optimizing the CUMS paradigm

**FIGURE 1:**
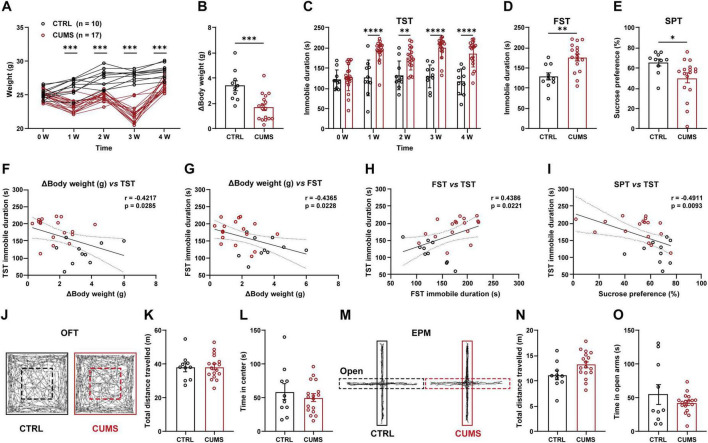
Phenotyping behavioral and homeostatic changes with the optimized CUMS in mice. **(A)** Changes in mouse body weight during 4-week CUMS exposure in mice. **(B)** Body weight reduction following 4-week CUMS. **(C–E)** The CUMS increased TST immobile duration **(C)**, FST immobile duration (**D**) and reduced sucrose preference (**E**) in mice. **(F–I)** Correlation coefficient analysis of multiple depressive phenotypes. **(J)** Representative track plots of the CTRL and CUMS mice in the OFT. **(K,L)** Quantification of the total distance travelled (**K**) and time in center (**L**) in the OFT. **(M)** Representative track plots of the CTRL and CUMS mice in the EPM. **(N,O)** Quantification of the total distance travelled **(N)** and time in open arms **(O)** in the EPM. Data are presented as the mean ± SEM; *P < 0.05, **P < 0.01, ***P < 0.001, ****P < 0.0001, Two-way ANOVA statistical analysis (**A**) or two-tailed unpaired Student’s *t*-test.

The CUMS paradigm is a widely employed animal model of depressive disorders that highly manifests the face, predictive, and construct validity of the depression disorder. The CUMS protocol was optimized to minimize the individual variation by combining short-term stressors with long-term stressors (See section “2 Materials and methods” and Table 1). Throughout the 4-week CUMS period, changes in body weight were continuously monitored. After 1-week of CUMS exposure, CUMS and control (CTRL) mice showed a significant difference in body weight, reaching its peak at 3 weeks and remaining significant even following the cessation of the CUMS (Figure 1A). Consistent with the weight loss observed in patients (Rice et al., 2019), mice that exposed to CUMS demonstrated a marked reduction in body weight gain, showing approximate 1.9 g reduction compared to CTRL mice (Figure 1B). As expected, CUMS exposure stably induced anhedonia- and despair-like behaviors, in the SPT, TST, and FST. Coinciding with the observed changes in body weight, differences in immobility during the TST reached significance after 1-week CUMS and persisted until the conclusion of the 4-week CUMS (Figure 1C). The immobile duration in the FST was increased from mean 128.6 s in CTRL mice to mean 176 s in CUMS mice (Figure 1D). In the SPT, the preference to sucrose was decreased from mean 65.57% in CTRL group to mean 49.85% in CUMS group (Figure 1E). The relevance between homeostasis and anhedonia-/despair-like behaviors was further supported, as evidenced by correlation coefficient analysis of the body weight gain with SPT and TST/FST performance (Figures 1F–I). Importantly, CUMS had no significant effects on the locomotion ability, as demonstrated by the insignificant changes of total distance moved in both the OFT and EPM test (Figures 1J, K, M, N). Moreover, neither the duration in the center nor in open arms was altered by the CUMS exposure, suggesting that anxiety-like behaviors are unaffected (Figures 1L, O). These results indicate that the optimized CUMS paradigm primarily contributes to the development of despair-like and anhedonia-like depressive phenotypes rather than anxiety-like phenotypes.

### 3.2 CUMS increases ovBNST activity in response to acute stress

**FIGURE 2:**
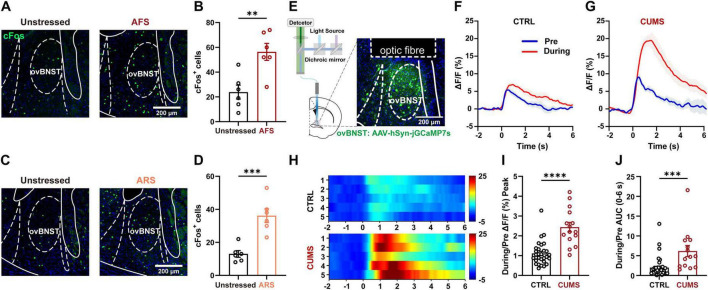
The ovBNST neuronal activity in naïve and CUMS mice in response to acute stress. **(A,B)** Representative images of cFos staining **(A)** and bar graphs displaying increased cFos-positive cell numbers in the ovBNST of mice subjected to AFS **(B**, *n* = 6). **(C,D)** Representative images of cFos staining **(C)** and bar graphs displaying increased cFos-positive cell numbers in the ovBNST of mice subjected to ARS (**D**, *n* = 6). **(E)** Schematic diagram of the fiber photometry and representative images displaying the viral expression and optical fiber implantation in the ovBNST. **(F–H)** Representative traces **(F,G)** and heatmaps **(H)** of the GCaMP fluorescence in the CTRL and CUMS mice responding to AFS. **(I,J)** Bar graphs showing quantification of GCaMP ΔF/F (%) Peak **(I)** and AUC (0–6 s) **(J)** in the ovBNST of CTRL and CUMS mice in responding to AFS (*n* = 6). Data are presented as the mean ± SEM, **P < 0.01, ***P < 0.001, ****P < 0.0001, two-tailed unpaired Student’s *t*-test. Scale bar: 200 μm.

Given that the BNST serves as a hub for mediating negative emotional states, neuronal activity within this region was investigated in both naïve and CUMS mice in response to acute stress. A typical immediately early gene (IEG), cFos, was chosen to indicate the neuronal activation during stress. Both acute forced swimming (AFS) and acute restraint stress (ARS) elevated the expression level of cFos by approximately two folds in the BNST, especially within the ovBNST subpopulation (Figures 2A–D). Considering the decay kinetics of cFos, *in vivo* imaging using fiber photometry was further employed to analyze time-resolved neuronal responses to AFS. AAVs carrying GCaMP were injected into the ovBNST, followed by optical fiber implantation above the same site (Figure 2E). Consistent with cFos staining, ovBNST neurons were strongly activated when the mouse was firstly placed in the water during the AFS (Figure 2F). Notably, compared with naïve mice, CUMS mice exhibited robust increases in both the peak and AUC during the AFS (Figures 2F–J).

### 3.3 Robust *cnr1* mRNA expression in mPFC inputs to the ovBNST

**FIGURE 3:**
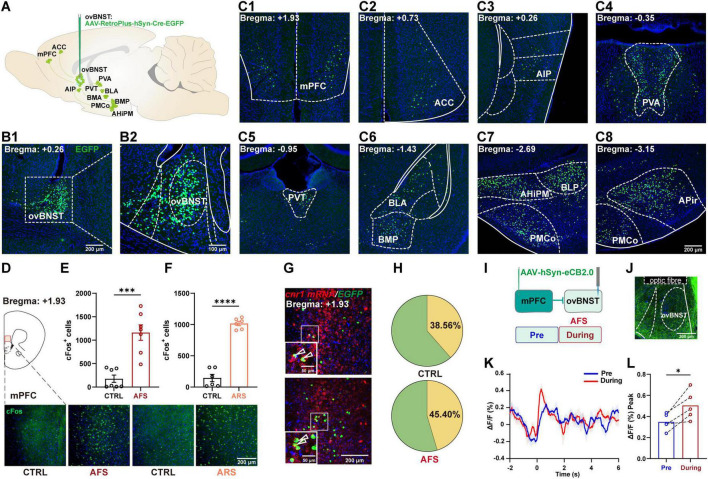
The *cnr1* mRNA is abundantly expressed in the mPFC-ovBNST projection and acute stress increases endocannabinoid release in the ovBNST. **(A)** Schematic showing the retrograde tracing of upstream nuclei with AAV-Retro Plus-hSyn-Cre-EGFP infusion in the ovBNST. **(B1,B2)** Representative overview (**B1**) and enlarged view (**B2**) illustrating the injection site in the ovBNST. **(C1–C8)** Representative images showing retrogradely labeled neurons distributed in the upstream nuclei including the mPFC **(C1)**, ACC **(C2)**, AIP **(C3)**, PVA **(C4)**, PVT **(C5)**, BLA **(C6)**, BMP **(C6)**, BLP **(C7)**, AHiPM **(C7)**, PMCO **(C7)**, and APir **(C8)**. **(D)** Illustrative representation of cFos staining in the mPFC. **(E,F)** Quantification of cFos cells in the mPFC of mice exposed to the AFS (**E**, *n* = 7) and ARS (**F**, *n* = 6). **(G)** Representative images of *cnr1* positive mPFC neurons projecting to the ovBNST. **(H)** Pie chart depicting the percentage of *cnr1*^+^/EGFP^+^ cells in the ovBNST (*n* = 5). **(I,J)** Schematic **(I)** and representative **(J)** images showing the injection of AAV-hSyn-eCB2.0 into the mPFC and implantation of recording fiber above the ovBNST. **(K)** Representative traces of the eCB2.0 fluorescence in the ovBNST of naïve mice in responding to AFS. **(L)** Bar graphs showing quantitative statistics of the endocannabinoid signals ΔF/F (%) Peak (*n* = 5). Data are presented as the mean ± SEM; *P < 0.05, ***P < 0.001, ****P < 0.0001, two-tailed unpaired or paired Student’s *t*-test. Scale bars: 200 μm for overviews and 100 μm or 50 μm for enlarged views.

To investigate upstream inputs mediating ovBNST neuronal responses to stress, a retrograde viral tracer carrying GFP tags (Retro-GFP) was infused into the ovBNST to map its afferent connections (Figures 3A, B). In accordance with previous report that the BNST is positioned at the hub of stress regulation network, GFP positive neurons were observed throughout the brain, particularly accumulated in the cortex, thalamus, and amygdala, including the mPFC, anterior cingulate cortex (ACC), posterior agranular insular cortex (AIP), anterior nucleus of paraventricular thalamus (PVA), paraventricular thalamus (PVT), basolateral amygdala (BLA), posterior basomedial amygdaloid nucleus (BMP), posterior basolateral amygdaloid nucleus (BLP), posteromedial amygdalohippocampal area (AHiPM), posteromedial cortical amygdaloid area (PMCo), and anterior piriform cortex (APir) (Figure 3C). Among these regions, the mPFC was activated by AFS and ARS, as indicated by pronounced cFos levels (Figures 3D–F). Moreover, according to the Allen ISH Data Reference Atlas, the mPFC exhibits abundant expression of *cnr1* mRNA which encodes the CB1R protein. The molecular identity of retrograded traced neurons was characterized with an RNAscope *cnr1* probe. Approximately 38.56% and 45.40% of the Retro-GFP labeled neurons were positive for *cnr1* in the mPFC of CTRL and AFS mice, respectively (Figures 3G, H). These findings indicate that CB1Rs are expressed in mPFC neurons projecting to the ovBNST, raising the possibility that the ECS may modulate the mPFC–ovBNST pathway through presynaptic mechanisms.

### 3.4 Acute stress increases endocannabinoid signaling in mPFC projection in the ovBNST

To investigate the response of mPFC^*CB*1*R*^-ovBNST pathway to acute stress, an recording optical fiber was implanted above the ovBNST following the infusion of endocannabinoid probe eCB2.0 in the mPFC to measure the endocannabinoid release from mPFC-ovBNST projection (Figures 3I, J). The AFS to naïve mice significantly increased the fluorescent intensity of eCB2.0 in a time-resolved manner, with the peak intensity rising from mean 0.35% pre the AFS to 0.51% during the AFS (Figures 3K, L). These results indicate that acute stress increases endocannabinoid signaling in the mPFC-ovBNST projection of naïve mice.

### 3.5 CB1R blockade in naïve mouse ovBNST induces depressive-like behaviors

**FIGURE 4:**
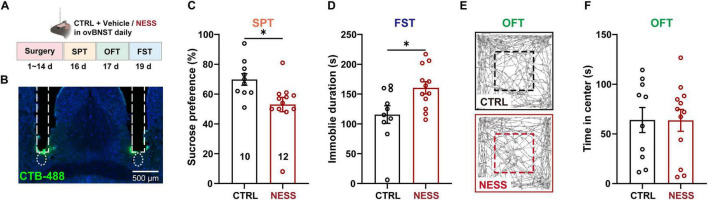
NESS-induced CB1R blockade in the ovBNST reduces sucrose preference and increases immobility in the FST. **(A)** Experimental diagram illustrating the pharmacological manipulation of CB1R and behavioral tests. **(B)** Representative images showing bilateral implantation of cannula above the ovBNST. **(C,D)** Analysis of sucrose preference (%) in the SPT **(C)** and immobile duration in the FST **(D)** following NESS infusion into the ovBNST. **(E,F)** Representative track plots (**E**) and time in center **(F)** in the OFT of the mice receiving infusion of vehicle or NESS into the ovBNST. Data are presented as the mean ± SEM; *P < 0.05, two-tailed unpaired Student’s *t*-test. Scale bar: 500 μm.

As the BNST occupies a pivotal position in the threats monitoring network, the ECS in the ovBNST likely mediates the stress responses. To investigate its role in stress coping, a pair of cannulae was bilaterally implanted above the ovBNST to infuse CB1R antagonists before depressive behavioral tests in naïve mice (Figure 4A). The locations of cannula tips were *post hoc* verified by fluorescent dye CTB-488 infusion (Figure 4B). To dissect the mechanistic basis of CB1R modulation in the ovBNST, two pharmacologically distinct antagonists were employed: a neutral antagonist NESS that blocks ligand binding without intrinsic activity and an antagonist/inverse agonist AM251 that blocks CB1R and suppresses constitutive receptor signaling. As evidenced by the SPT, CB1R blockade in the ovBNST using the CB1R neutral antagonist NESS resulted in a significant reduction of sucrose preference by 12.13% (Figure 4C). Meanwhile, compared to the CTRL group, the immobility duration was increased in the NESS group during the FST (Figure 4D). These findings suggest that the physiological endocannabinoid binding is essential for stress resilience. However, intra-ovBNST administration of the CB1R antagonist/inverse agonist AM251 marginally decreased sucrose preference without reaching statistical significance (Supplementary Figure 1A). The immobile duration in the FST was prolonged from 122.66 s to 168.5 s (Supplementary Figure 1B). Neither the travel distance nor the duration spent in the center zone was altered by CB1R blockage (Figures 4E, F and Supplementary Figures 1C, D). Together, loss-of-function interventions demonstrated that impairments of ovBNST ECS induced depressive rather than anxious phenotypes in naïve mice, suggesting that the normal endocannabinoid signaling in the ovBNST is necessary for protecting naïve mice from developing depression-like behaviors.

### 3.6 Increased endocannabinoid signaling in the ovBNST of CUMS mice alleviates depression

**FIGURE 5:**
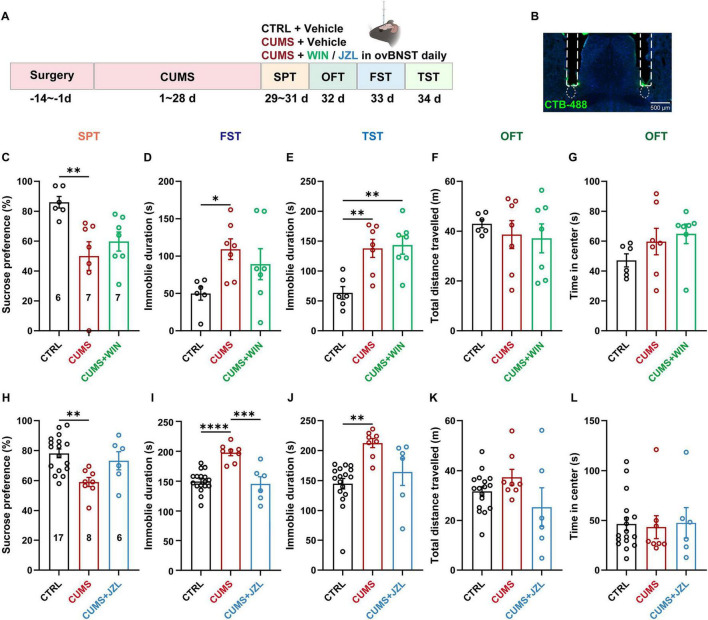
Increased endocannabinoid signaling in the ovBNST ameliorates CUMS induced depressive-like behaviors. **(A)** Experimental diagram illustrating the CUMS modeling and pharmacological manipulation of CB1R with the CB1R agonist WIN or MAGL inhibitor JZL-184 administration in the ovBNST. **(B)** Representative images showing bilateral implantation of cannula above the ovBNST. **(C–G)** Analysis of sucrose preference (%) in the SPT **(C)**, immobile duration in the FST **(D)** and TST **(E)**, total distance travelled **(F)** and time in center **(G)** in the OFT following the CB1R agonist WIN infusion into the ovBNST in CTRL and CUMS mice. **(H–L)** Data corresponding to **(C–G)**, but for the mice subjected to the MAGL inhibitor JZL-184 infusion. Data are presented as the mean ± SEM; *P < 0.05, **P < 0.01, ***P < 0.001, ****P < 0.0001, data were analyzed using one-way ANOVA followed by *Tukey-Kramer post hoc* test for pairwise comparisons. Scale bar: 500 μm.

To assess whether activation of CB1Rs in inputs to the ovBNST can ameliorate depressive phenotypes under pathological conditions, a similar assay was conducted by infusing the CB1R synthetic agonist WIN into the ovBNST of mice subjected to the CUMS (Figures 5A, B). Unexpectedly, intra-ovBNST administration of WIN failed to alleviate depression-like behaviors or anxiety-like behaviors (Figures 5C–G). While a slightly WIN-induced enhancement of sucrose preference negated the distinguishment between the WIN and CTRL groups, there was no statistically significant variance between the WIN and CUMS groups (Figure 5C). Subsequently, the MAGL inhibitor JZL-184 was administered into the ovBNST of CUMS mice to upregulate local levels of endocannabinoid 2-AG. Intriguingly, bilateral ovBNST infusion of JZL-184 in CUMS mice significantly reduced immobility in the FST, while only trending toward increased sucrose preference and decreased TST immobility (*P* = 0.0713), demonstrating partial improvements in the despair-like behaviors (Figures 5H–J). Furthermore, both locomotor activities and anxiety-like behaviors remained unchanged (Figures 5F, G and K, L). These results suggest that increased endocannabinoid signaling in ovBNST produces antidepressant effects in CUMS mice.

### 3.7 Alterations in the mPFC *cnr1* mRNA level and ovBNST CB1R protein level post CUMS exposure

**FIGURE 6:**
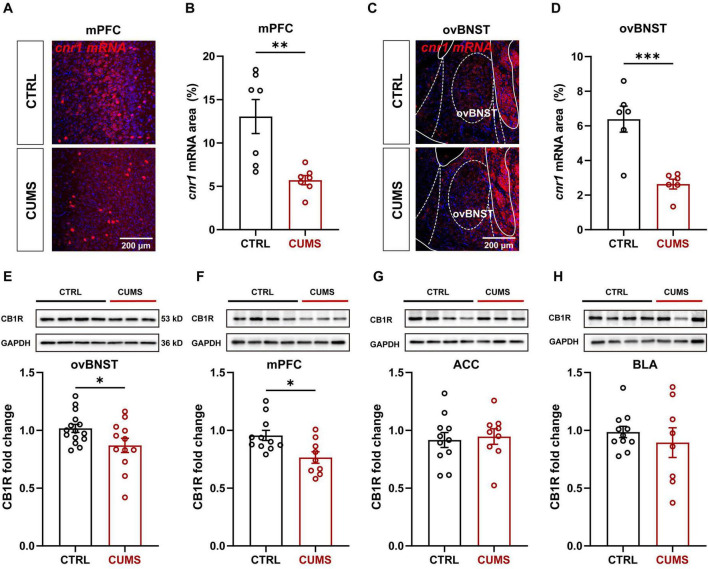
Alterations in *cnr1* mRNA and CB1R protein levels in the mPFC-ovBNST pathway of CUMS mice. **(A)** Representative images showing alterations in *cnr1* mRNA levels in the mPFC following CUMS exposure. **(B)** Quantification of *cnr1* mRNA levels in the mPFC of CTRL and CUMS mice (*n* = 7). **(C)** Representative images showing alterations in *cnr1* mRNA levels in the ovBNST following CUMS exposure. **(D)** Quantification of *cnr1* mRNA levels in the ovBNST of CTRL and CUMS mice (*n* = 6). **(E–H)** Representative images showing alterations in CB1R protein levels in the mPFC following CUMS exposure and quantification of CB1R protein levels in the ovBNST **(E)**, mPFC **(F)**, ACC **(G)**, and BLA **(H)** in CTRL and CUMS mice (ovBNST, CTRL, *n* = 15, CUMS, *n* = 12; mPFC, CTRL, *n* = 11, CUMS, *n* = 9; ACC, CTRL, *n* = 11, CUMS, *n* = 9; BLA, CTRL, *n* = 11, CUMS, *n* = 8). Data are presented as the mean ± SEM; *P < 0.05, **P < 0.01, ***P < 0.001, two-tailed unpaired Student’s *t*-test. Scale bar: 200 μm.

Next, the transcriptional and translational levels of CB1Rs were assessed using the RNAscope and Western blotting, respectively. Exposure to the CUMS significantly decreased the level of *cnr1* mRNA in both the mPFC and ovBNST by over two folds (Figures 6A–D). Given that CB1Rs are localized presynaptically, the downregulation of *cnr1* mRNA in the ovBNST suggests that the CUMS reduces CB1R protein expression in ovBNST-innervating downstream. In accordance with the observed reduction of somatic *cnr1* mRNA expression in the mPFC, the presynaptic CB1R protein level after the CUMS was downregulated in the ovBNST (Figure 6E), indicating that the reduced expression of CB1R proteins in ovBNST axonal terminals may at least partially originate from the mPFC. Concurrently, the expression level of CB1R in the mPFC was also reduced in the CUMS group, implying that CUMS may modulate the plasticity of the mPFC through its influence on upstream CB1R innervation (Figure 6F). Interestingly, no significant alterations in CB1R expression were detectable in other regions projecting to the ovBNST, including the ACC and BLA (Figures 6G, H). These findings indicate that the ECS is crucial for the mediation of depression-like behaviors potentially through the mPFC-ovBNST pathway.

## 4 Discussion

Stress significantly impacts a range of physiological and psychological processes, within which framework the ECS has emerged as a crucial stress-inhibitory regulator (Battista et al., 2012; deRoon-Cassini et al., 2020). The present study uncovered the action of the ECS on modulating the ovBNST neuronal activity and its role in regulating the depressive phenotypes following the CUMS. Both the ovBNST and mPFC were activated by acute stress, with increased release of endocannabinoid in the former region. Furthermore, blockage of the ovBNST CB1Rs heightened the depression level in naïve mice, whereas enhancement of the ECS partially ameliorated depressive phenotypes in mice subjected to the CUMS. These observations suggest a reliance on the mPFC-ovBNST pathway, as elucidated by the integration of retrograde viral tracing with CB1R assay. Collectively, our study identified potential neural circuit and molecular target underlying the pathogenesis of depressive disorders induced by chronic stress.

### 4.1 The CUMS induced depressive behavioral and homeostatic phenotypes

The CUMS represents a reliable approach for modeling depression in rodents. It was optimized in this study to minimize the variability by integrating short-term stressors with long-term stressors, thereby reproducing the classical depressive phenotypes, such as anhedonia- and despair-like behaviors, as well as body weight loss. As revealed in a number of clinical studies, depressive episodes are typically associated with weight loss, which can be reversed by effective antidepressants (Uher et al., 2011). Notably, consistent individual performance across the FST, TST, and SPT correlated with body weight change, suggesting variability in stress sensitivity among CUMS mice. This behavioral and physiological correlation may reflect a continuum of susceptibility and resilience, as reported in both rodents and humans (Pantoja-Urbán et al., 2024; Wang and Zhang, 2017). As a widely distributed stress responder in the brain, the ECS may contribute to stress resilience as a potential mediator of synaptic transmission in the mPFC and BLA (Worley et al., 2018). Both genetic ablation and pharmacologic antagonization of the CB1R exaggerated behavioral responses to stress (Morena et al., 2016; Beyer et al., 2010; Hill et al., 2011). Whereas, facilitation of endocannabinoid signaling mitigated the impact of both acute and chronic stress on mood and behavioral changes (Morena et al., 2016).

### 4.2 Roles of the BNST ECS in stress processing

The BNST, composed of multiple heterogenous subnuclei, serves as a critical hub for threat assessment, with the ovBNST and other subregions exerting opposite effects in modulating diverse features of anxiety states (Kim et al., 2013). The increased fluorescence of the eCB2.0 reflected an elevation in the endocannabinoid release from the CB1R enriched ovBNST elicited by acute stress. Individuals with major depressive disorder have been found to exhibit changes in the circulating levels of AEA and 2-AG (Morena et al., 2016). The dynamic regulation of these endocannabinoids by stress occurs in a bidirectional manner. Generally, stress exposure reduces AEA and increases 2-AG levels throughout most brain regions (Morena et al., 2016; Marcus et al., 2020). Moreover, AEA is characterized as a “tonic” signal, whereas 2-AG functions as a ‘phasic’ signal that mediates various forms of synaptic plasticity (Morena et al., 2016). Collectively, these findings suggest that 2-AG might be responsible for the rapid increase in the eCB2.0 fluorescence at the early stage of acute stress coping. Previous studies have reported that ARS and foot shock mildly increased 2-AG within the mPFC and periaqueductal gray (PAG), respectively (Hill et al., 2011; Hohmann et al., 2005). However, due to the similar affinity of both AEA and 2-AG for this probe (Dong et al., 2022), it is not feasible to distinguish the exact ligand involved in this process due to methodological limitations. Sensors specifically probing the AEA or 2-AG would benefit the address of this question. Although both antagonists elicited depressive-like behaviors in naïve mice to some extent, the neutral antagonist NESS produced statistically significant effects in the SPT and FST, whereas the antagonist/inverse agonist AM251 showed variable efficacy. This divergence implies that loss of constitutive CB1R signaling through inverse agonism might be partially compensated through alternative mechanisms. However, additional research is necessary to confirm this hypothesis. More importantly, the consistent manifestation of depressive phenotypes with both ligands underscores the critical role of intact CB1R signaling in conferring stress resilience.

### 4.3 JZL-184 ameliorates depression-like behaviors under more physiologically relevant conditions

Intra-ovBNST infusion of both CB1R agonist WIN and cannabinoid hydrolase inhibitor JZL-184 can enhance the endocannabinoid signaling in the ovBNST, leading to partial amelioration of depressive-like behaviors induced by the CUMS. Nonetheless, the antidepressant effects exhibit notable differences as a result of their different action mechanisms. Only JZL-184 demonstrated efficacy in alleviating despair-like behaviors, particularly effective in diminishing the FST immobility. Although neither compound produced a statistically significant improvement in anhedonia-like behaviors, the SPT performance of CUMS mice receiving JZL-184 treatment was comparatively similar to that of the CTRL group. As a synthetic cannabinoid receptor agonist, WIN directly activates CB1Rs with a rapid onset of action but shorter duration. However, chronic use of WIN may lead to desensitization and downregulation of CB1Rs, which can reduce efficacy over time and potentially induce side effects, including cognitive and motor impairments (Sim-Selley and Billy, 2002; Moranta et al., 2009). Considering that the CUMS exposure also downregulated CB1R in the ovBNST, repeated infusion of WIN may fail to improve or even worsen the depressive phenotypes due to dysfunctional CB1Rs. On the other hand, JZL-184 is a potent and selective inhibitor of MAGL, leading to increased levels of endogenous 2-AG levels by inhibiting its breakdown (Kano et al., 2009). By maintaining elevated 2-AG levels, JZL-184 has a more prolonged effect without receptor desensitization, making it a safer approach suitable for long-term management of mood disorders with fewer adverse effects and less impairment of CB1Rs (Moranta et al., 2009; Sepers et al., 2024). While our study focused on 2-AG modulation, the role of AEA and its hydrolytic enzyme FAAH is also of great interest. Chronic stress has been reported to boost FAAH activity, thereby reducing AEA levels (deRoon-Cassini et al., 2020; Patel and Hillard, 2008). Reversing FAAH hyperactivity can mitigate chronic stress effects (Segev et al., 2018), indicating that targeting FAAH may be a promising therapeutic strategy for stress-related disorders. Future studies could explore the potential of simultaneous inhibition of FAAH and MAGL to enhance endocannabinoid signaling for treating depression-like behaviors.

### 4.4 The CUMS restructured the ECS to cause BNST hypersensitivity

Vulnerability to stressors is a prevalent characteristic of depression disorders. Compared with naïve mice, increased Ca^2+^ signals in the ovBNST were detected when the depressive mice suffered from swimming in the ice water, suggesting that the CUMS leads to hyperactivity of the ovBNST in response to acute stress. Considering that acute stress induced the release of endocannabinoid in the ovBNST, it can be inferred that the CUMS modified the sensitivity of the ovBNST to threats via the modulation of the ECS. Clinical research has reported that the human BNST is hyperactive during re-exposure to previously experienced trauma (Awasthi et al., 2020). Numerous studies have reported that the stress-sensitive ECS is a key modulator of synaptic plasticity which is crucial for individuals to recalibrate stress responses based on prior experience. Intra-BNST infusion of a CB1R agonist efficiently suppressed the mPFC glutamatergic innervation of the BNST (Massi et al., 2008). In contrast, the infusion of a CB1R antagonist into the BNST blocked the restraint stress elicited long-term potentiation (Glangetas et al., 2013). Therefore, the CUMS likely remodeled the ECS to influence the synaptic plasticity of the ovBNST through both the release of endocannabinoid ligands and the dynamics of CB1Rs.

### 4.5 Study limitations

Our study has revealed the crucial role of the ECS within the mPFC-ovBNST pathway in the pathogenesis of depressive disorder; however, there are some limitations. Our study focused primarily on male mice, which may restrict the generalizability of our findings to female subjects. Given the well-established influence of sex on stress vulnerability and documented variations in the distribution of ECS across brain regions between males and females (Bale and Epperson, 2015; Craft et al., 2013; Bangasser et al., 2010; Blanton et al., 2021), future studies should include both sexes to ensure a more comprehensive and generalizable understanding of ECS-mediated stress responses and depression-like behaviors. Although upregulating endocannabinoid signaling in the ovBNST using JZL-184 alleviated depressive phenotypes induced by the CUMS exposure, the effects were particularly significant in the FST performance. This suggests a possible dose-dependent effect, where increasing the dosage of JZL-184 may be more effective in alleviating anhedonia- and despair-like behaviors, which can be assessed using the SPT and TST. In addition to pharmacological intervention, optogenetic manipulation offers a more precise method for enhancing endocannabinoid signaling and could serve as an alternative approach in future research. Another limitation is the lack of investigation into whether overexpression of CB1R within this pathway can rescue depressive phenotypes or enhance resilience to CUMS exposure, an aspect that holds particular significance from a preventive medicine perspective.

## 5 Conclusion

In summary, the results suggest that the ECS enriched in the ovBNST may contribute to stress-related synaptic plasticity, potentially involving CB1Rs expressed afferent projections such as those from the mPFC, which underlies the CUMS induced pathogenesis of depressive phenotypes. The present study may shed lights on the path toward developing ECS-based therapeutic strategies for managing chronic stress-related mood disorders.

## Data Availability

The raw data supporting the conclusions of this article will be made available by the authors, without undue reservation.

## Supplementary material

Supplementary Figure 1 AM251-mediated CB1R blockade in the ovBNST prolongs immobility duration in the FST. **(A,B)** Analysis of sucrose preference (%) in the SPT (**A**) and immobile duration in the FST (**B**) following AM251 infusion into the ovBNST. **(C,D)** Representative track plots (**C**) and time in center (**D**) in the OFT of the mice receiving infusion of vehicle or AM251 into the ovBNST. Data are presented as the mean ± SEM; ***P* < 0.01, two-tailed unpaired Student’s *t*-test.

## References

[B1] Avery S. N. Clauss J. A. Blackford J. U. (2016). The human BNST: Functional role in anxiety and addiction. *Neuropsychopharmacology* 41 126–141. 10.1038/npp.2015.185 26105138 PMC4677124

[B2] Awasthi S. Pan H. LeDoux J. Cloitre M. Altemus M. McEwen B. (2020). The bed nucleus of the stria terminalis and functionally linked neurocircuitry modulate emotion processing and HPA axis dysfunction in posttraumatic stress disorder. *Neuroimage Clin.* 28:102442. 10.1016/j.nicl.2020.102442 33070099 PMC7569227

[B3] Bale T. L. Epperson C. N. (2015). Sex differences and stress across the lifespan. *Nat. Neurosci.* 18 1413–1420. 10.1038/nn.4112 26404716 PMC4620712

[B4] Bangasser D. Curtis A. Reyes B. Bethea T. Parastatidis I. Ischiropoulos H. (2010). Sex differences in corticotropin-releasing factor receptor signaling and trafficking: Potential role in female vulnerability to stress-related psychopathology. *Mol. Psychiatry* 15 877, 896–904. 10.1038/mp.2010.66 20548297 PMC2935505

[B5] Battista N. Di Tommaso M. Bari M. Maccarrone M. (2012). The endocannabinoid system: An overview. *Front. Behav. Neurosci.* 6:9. 10.3389/fnbeh.2012.00009 22457644 PMC3303140

[B6] Beyer C. Dwyer J. Piesla M. Platt B. Shen R. Rahman Z. (2010). Depression-like phenotype following chronic CB1 receptor antagonism. *Neurobiol. Dis.* 39 148–155. 10.1016/j.nbd.2010.03.020 20381618

[B7] Blanton H. Barnes R. McHann M. Bilbrey J. Wilkerson J. Guindon J. (2021). Sex differences and the endocannabinoid system in pain. *Pharmacol. Biochem. Behav.* 202:173107. 10.1016/j.pbb.2021.173107 33444598 PMC8216879

[B8] Calhoon G. G. Tye K. M. (2015). Resolving the neural circuits of anxiety. *Nat. Neurosci.* 18 1394–1404. 10.1038/nn.4101 26404714 PMC7575249

[B9] Craft R. M. Marusich J. A. Wiley J. L. (2013). Sex differences in cannabinoid pharmacology: A reflection of differences in the endocannabinoid system? *Life Sci.* 92 476–481. 10.1016/j.lfs.2012.06.009 22728714 PMC3492530

[B10] Crestani C. C. Alves F. H. Gomes F. V. Resstel L. B. Correa F. M. Herman J. P. (2013). Mechanisms in the bed nucleus of the stria terminalis involved in control of autonomic and neuroendocrine functions: A review. *Curr. Neuropharmacol.* 11 141–159. 10.2174/1570159X11311020002 23997750 PMC3637669

[B11] deRoon-Cassini T. Stollenwerk T. Beatka M. Hillard C. (2020). Meet your stress management professionals: The endocannabinoids. *Trends Mol. Med.* 26 953–968. 10.1016/j.molmed.2020.07.002 32868170 PMC7530069

[B12] Dong A. He K. Dudok B. Farrell J. Guan W. Liput D. (2022). A fluorescent sensor for spatiotemporally resolved imaging of endocannabinoid dynamics in vivo. *Nat .Biotechnol.* 40 787–798. 10.1038/s41587-021-01074-4 34764491 PMC9091059

[B13] GBD 2019 Mental Disorders Collaborators. (2022). Global, regional, and national burden of 12 mental disorders in 204 countries and territories, 1990-2019: A systematic analysis for the global burden of disease study 2019. *Lancet Psychiatry* 9 137–150. 10.1016/S2215-0366(21)00395-3 35026139 PMC8776563

[B14] Glangetas C. Georges F. (2016). Pharmacology of the bed nucleus of the stria terminalis. *Curr. Pharmacol. Rep.* 2 262–270. 10.1007/s40495-016-0077-7

[B15] Glangetas C. Girard D. Groc L. Marsicano G. Chaouloff F. Georges F. (2013). Stress switches cannabinoid type-1 (CB1) receptor-dependent plasticity from LTD to LTP in the bed nucleus of the stria terminalis. *J. Neurosci.* 33 19657–19663. 10.1523/JNEUROSCI.3175-13.2013 24336729 PMC6618762

[B16] Gomes-de-Souza L. Bianchi P. Costa-Ferreira W. Tomeo R. Cruz F. Crestani C. (2021). CB1 and CB2 receptors in the bed nucleus of the stria terminalis differently modulate anxiety-like behaviors in rats. *Prog. Neuropsychopharmacol. Biol. Psychiatry* 110:110284. 10.1016/j.pnpbp.2021.110284 33609604

[B17] Han J. Kesner P. Metna-Laurent M. Duan T. Xu L. Georges F. (2012). Acute cannabinoids impair working memory through astroglial CB1 receptor modulation of hippocampal LTD. *Cell* 148 1039–1050. 10.1016/j.cell.2012.01.037 22385967

[B18] Heshmati M. Russo S. J. (2015). Anhedonia and the brain reward circuitry in depression. *Curr. Behav. Neurosci. Rep.* 2 146–153. 10.1007/s40473-015-0044-3 26525751 PMC4626008

[B19] Hill M. N. Hillard C. J. Mcewen B. S. (2011). Alterations in corticolimbic dendritic morphology and emotional behavior in cannabinoid CB1 receptor-deficient mice parallel the effects of chronic stress. *Cereb. Cortex* 21 2056–2064. 10.1093/cercor/bhq280 21263035 PMC3155602

[B20] Hohmann A. Suplita R. Bolton N. Neely M. Fegley D. Mangieri R. (2005). An endocannabinoid mechanism for stress-induced analgesia. *Nature* 435 1108–1112. 10.1038/nature03658 15973410

[B21] Huang W.-J. Chen W.-W. Zhang X. (2016). Endocannabinoid system: Role in depression, reward and pain control (Review). *Mol. Med. Rep.* 14 2899–2903. 10.3892/mmr.2016.5585 27484193 PMC5042796

[B22] Iversen L. (2003). Cannabis and the brain. *Brain* 126 1252–1270. 10.1093/brain/awg143 12764049

[B23] Kano M. Ohno-Shosaku T. Hashimotodani Y. Uchigashima M. Watanabe M. (2009). Endocannabinoid-mediated control of synaptic transmission. *Physiol. Rev.* 89 309–380. 10.1152/physrev.00019.2008 19126760

[B24] Kilaru A. Chapman K. D. (2020). The endocannabinoid system. *Essays Biochem.* 64 485–499. 10.1042/EBC20190086 32648908

[B25] Kim S. Adhikari A. Lee S. Marshel J. Kim C. Mallory C. (2013). Diverging neural pathways assemble a behavioural state from separable features in anxiety. *Nature* 496 219–223. 10.1038/nature12018 23515158 PMC6690364

[B26] Lange M. Daldrup T. Remmers F. Szkudlarek H. Lesting J. Guggenhuber S. (2017). Cannabinoid CB1 receptors in distinct circuits of the extended amygdala determine fear responsiveness to unpredictable threat. *Mol. Psychiatry* 22 1422–1430. 10.1038/mp.2016.156 27698427

[B27] Lebow M. A. Chen A. (2016). Overshadowed by the amygdala: The bed nucleus of the stria terminalis emerges as key to psychiatric disorders. *Mol. Psychiatry* 21 450–463. 10.1038/mp.2016.1 26878891 PMC4804181

[B28] Maccarrone M. (2017). Metabolism of the endocannabinoid anandamide: Open questions after 25 years. *Front. Mol. Neurosci.* 10:166. 10.3389/fnmol.2017.00166 28611591 PMC5447297

[B29] Maccarrone M. (2020). Missing pieces to the endocannabinoid puzzle. *Trends Mol. Med.* 26 263–272. 10.1016/j.molmed.2019.11.002 31822395

[B30] Maccarrone M. Bab I. Bíró T. Cabral G. Dey S. Di Marzo V. (2015). Endocannabinoid signaling at the periphery: 50 years after THC. *Trends Pharmacol. Sci.* 36 277–296. 10.1016/j.tips.2015.02.008 25796370 PMC4420685

[B31] Marcus D. Bedse G. Gaulden A. Ryan J. Kondev V. Winters N. (2020). Endocannabinoid signaling collapse mediates stress-induced amygdalo-cortical strengthening. *Neuron* 105:1062–1076.e6. 10.1016/j.neuron.2019.12.024 31948734 PMC7992313

[B32] Massi L. Elezgarai I. Puente N. Reguero L. Grandes P. Manzoni O. (2008). Cannabinoid receptors in the bed nucleus of the stria terminalis control cortical excitation of midbrain dopamine cells in vivo. *J. Neurosci.* 28 10496–10508. 10.1523/JNEUROSCI.2291-08.2008 18923026 PMC6671338

[B33] Mechoulam R. Parker L. A. (2013). The endocannabinoid system and the brain. *Annu. Rev. Psychol.* 64 21–47. 10.1146/annurev-psych-113011-143739 22804774

[B34] Moranta D. Esteban S. Garcia-Sevilla J. A. (2009). Chronic treatment and withdrawal of the cannabinoid agonist WIN 55,212-2 modulate the sensitivity of presynaptic receptors involved in the regulation of monoamine syntheses in rat brain. *Naunyn. Schmiedebergs Arch. Pharmacol.* 379 61–72. 10.1007/s00210-008-0337-0 18709357

[B35] Morena M. Patel S. Bains J. Hill M. (2016). Neurobiological interactions between stress and the endocannabinoid system. *Neuropsychopharmacology* 41 80–102. 10.1038/npp.2015.166 26068727 PMC4677118

[B36] Nestler E. Barrot M. DiLeone R. Eisch A. Gold S. Monteggia L. (2002). Neurobiology of depression. *Neuron* 34 13–25. 10.1016/s0896-6273(02)00653-0 11931738

[B37] Pan B. Wang W. Zhong P. Blankman J. L. Cravatt B. F. Liu Q. S. (2011). Alterations of endocannabinoid signaling, synaptic plasticity, learning, and memory in monoacylglycerol lipase knock-out mice. *J. Neurosci.* 31 13420–13430. 10.1523/JNEUROSCI.2075-11.2011 21940435 PMC3371386

[B38] Pantoja-Urbán A. Richer S. Mittermaier A. Giroux M. Nouel D. Hernandez G. (2024). Gains and losses: Resilience to social defeat stress in adolescent female mice. *Biol. Psychiatry* 95 37–47. 10.1016/j.biopsych.2023.06.014 37355003 PMC10996362

[B39] Patel S. Hillard C. J. (2008). Adaptations in endocannabinoid signaling in response to repeated homotypic stress: A novel mechanism for stress habituation. *Eur. J. Neurosci.* 27 2821–2829. 10.1111/j.1460-9568.2008.06266.x 18588527 PMC2593941

[B40] Pertwee R. (1997). Pharmacology of cannabinoid CB1 and CB2 receptors. *Pharmacol. Ther.* 74 129–180. 10.1016/s0163-7258(97)82001-3 9336020

[B41] Pertwee R. G. (2001). Cannabinoid receptor ligands. *Tocris Rev.* 16 1–8.

[B42] Puente N. Cui Y. Lassalle O. Lafourcade M. Georges F. Venance L. (2011). Polymodal activation of the endocannabinoid system in the extended amygdala. *Nat. Neurosci.* 14 1542–1547. 10.1038/nn.2974 22057189

[B43] Rice F. Riglin L. Lomax T. Souter E. Potter R. Smith D. (2019). Adolescent and adult differences in major depression symptom profiles. *J. Affect. Disord.* 243 175–181. 10.1016/j.jad.2018.09.015 30243197

[B44] Russell S. Rachlin A. Smith K. Muschamp J. Berry L. Zhao Z. (2014). Sex differences in sensitivity to the depressive-like effects of the kappa opioid receptor agonist U-50488 in rats. *Bio.l Psychiatry* 76 213–222. 10.1016/j.biopsych.2013.07.042 24090794 PMC4476271

[B45] Segev A. Korem N. Mizrachi Zer-Aviv T. Abush H. Lange R. Sauber G. (2018). Role of endocannabinoids in the hippocampus and amygdala in emotional memory and plasticity. *Neuropsychopharmacology* 43 2017–2027. 10.1038/s41386-018-0135-4 29977073 PMC6098035

[B46] Sepers M. D. Woodard C. L. Ramandi D. Vecchiarelli H. A. Hill M. N. Raymond L. A. (2024). Effect of chronic upregulation of endocannabinoid signaling in vivo with JZL184 on striatal synaptic plasticity and motor learning in YAC128 Huntington disease mice. *bioRxiv* [Preprint]. 10.1101/2024.09.25.614804 bioRxiv:2024.09.25.614804. PMC12231859 40275705

[B47] Shen C.-J. Zheng D. Li K.-X. Yang J.-M. Pan H.-Q. Yu X.-D. (2019). Cannabinoid CB1 receptors in the amygdalar cholecystokinin glutamatergic afferents to nucleus accumbens modulate depressive-like behavior. *Nat. Med.* 25 337–349. 10.1038/s41591-018-0299-9 30643290

[B48] Sim-Selley L. J. M. Billy R. (2002). Effect of chronic administration of R-(+)-[2,3-Dihydro-5-methyl-3-[(morpholinyl)methyl]pyrrolo[1,2,3-de]-1,4-benzoxazinyl]-(1-naphthalenyl)methanone mesylate (WIN55,212-2) or delta(9)-tetrahydrocannabinol on cannabinoid receptor adaptation in mice. *J. Pharmacol. Exp. Ther.* 303 36–44. 10.1124/jpet.102.035618 12235230

[B49] Tsou K. Brown S. Sañudo-Peña M. Mackie K. Walker J. (1998). Immunohistochemical distribution of cannabinoid CB1 receptors in the rat central nervous system. *Neuroscience* 83 393–411. 10.1016/s0306-4522(97)00436-3 9460749

[B50] Uher R. Mors O. Hauser J. Rietschel M. Maier W. Kozel D. (2011). Changes in body weight during pharmacological treatment of depression. *Int. J. Neuropsychopharmacol.* 14 367–375. 10.1017/S1461145710000933 20716398

[B51] Valverde O. Torrens M. (2012). CB1 receptor-deficient mice as a model for depression. *Neuroscience* 204 193–206. 10.1016/j.neuroscience.2011.09.031 21964469

[B52] Wang X. Zhang Y. Wang X. Dai J. Hua R. Zeng S. (2020). Anxiety-related cell-type-specific neural circuits in the anterior-dorsal bed nucleus of the stria terminalis. *Sci. Bull. (Beijing)* 65 1203–1216. 10.1016/j.scib.2020.03.028 36659150

[B53] Wang Y. Zhang X. (2017). FAAH inhibition produces antidepressant-like efforts of mice to acute stress via synaptic long-term depression. *Behav. Brain Res.* 324 138–145. 10.1016/j.bbr.2017.01.054 28193523

[B54] Williford K. Taylor A. Melchior J. Yoon H. Sale E. Negasi M. (2023). BNST PKCδ neurons are activated by specific aversive conditions to promote anxiety-like behavior. *Neuropsychopharmacology* 48 1031–1041. 10.1038/s41386-023-01569-5 36941364 PMC10209190

[B55] Willner P. Muscat R. Papp M. (1992). Chronic mild stress-induced anhedonia: A realistic animal model of depression. *Neurosci. Biobehav. Rev.* 16 525–534. 10.1016/s0149-7634(05)80194-0 1480349

[B56] Willner P. Towell A. Sampson D. Sophokleous S. Muscat R. (1987). Reduction of sucrose preference by chronic unpredictable mild stress, and its restoration by a tricyclic antidepressant. *Psychopharmacology (Berl)* 93 358–364. 10.1007/BF00187257 3124165

[B57] World Health Organization (2017). *Depression and other common mental disorders: Global health estimates.* Geneva: World Health Organization.

[B58] World Health Organization (2021). *Mental health atlas 2020.* Geneva: World Health Organization.

[B59] Worley N. B. Hill M. N. Christianson J. P. (2018). Prefrontal endocannabinoids, stress controllability and resilience: A hypothesis. *Prog. Neuropsychopharmacol. Biol. Psychiatry* 85 180–188. 10.1016/j.pnpbp.2017.04.004 28392485 PMC6746235

[B60] Yang Y. Cui Y. Sang K. Dong Y. Ni Z. Ma S. (2018). Ketamine blocks bursting in the lateral habenula to rapidly relieve depression. *Nature* 554 317–322. 10.1038/nature25509 29446381

[B61] Zhang W. H. Zhang J. Y. Holmes A. Pan B. X. (2021). Amygdala circuit substrates for stress adaptation and adversity. *Biol. Psychiatry* 89 847–856. 10.1016/j.biopsych.2020.12.026 33691931

[B62] Zou S. Kumar U. (2018). Cannabinoid receptors and the endocannabinoid system: Signaling and function in the central nervous system. *Int. J. Mol. Sci.* 19:833. 10.3390/ijms19030833 29533978 PMC5877694

