# Supplementary figures and images for: Endocannabinoid-mediated regulation of depression in the ovBNST
Source: Front Neurosci. 2025 Jul 25;19:1629351. doi: 10.3389/fnins.2025.1629351 (PMC12331707; doi:10.3389/fnins.2025.1629351)

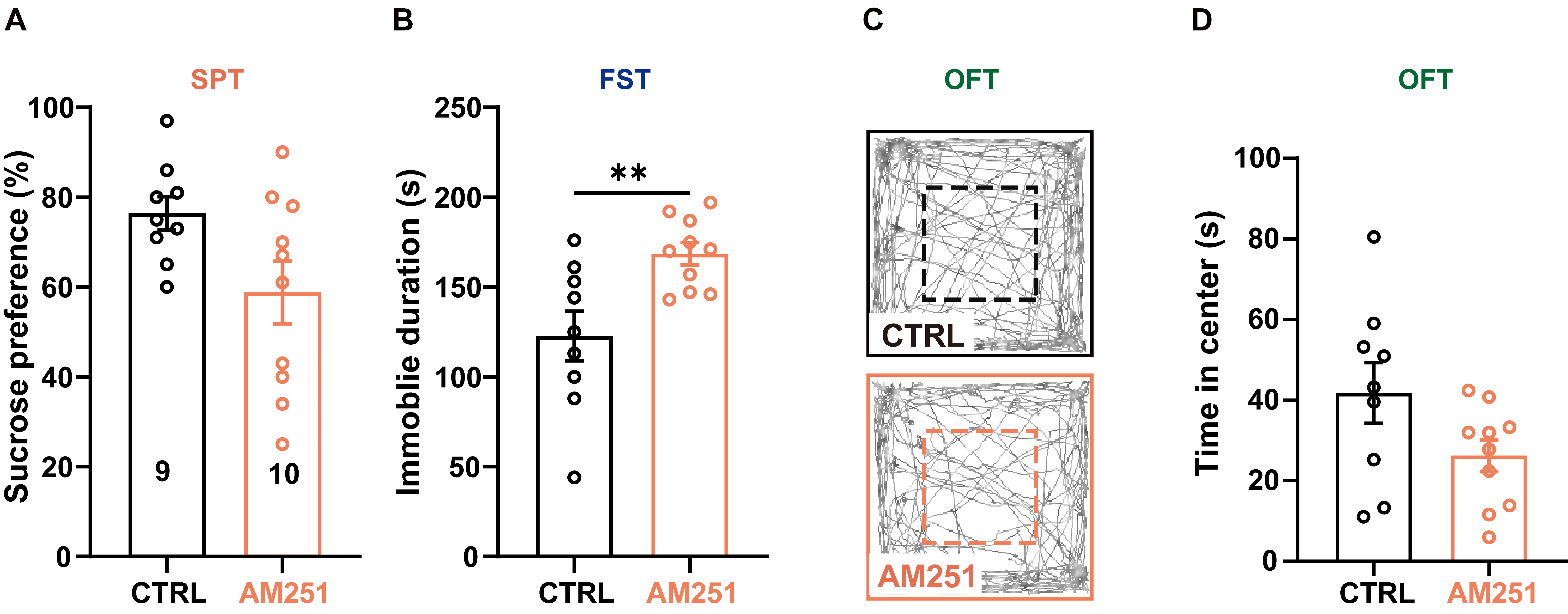

Supplement: Supplementary Figure 1 — AM251-mediated CB1R blockade in the ovBNST prolongs immobility duration in the FST. (A,B) Analysis of sucrose preference (%) in the SPT (A) and immobile duration in the FST (B) following AM251 infusion into the ovBNST. (C,D) Representative track plots (C) and time in center (D) in the OFT of the mice receiving infusion of vehicle or AM251 into the ovBNST. Data are presented as the mean ± SEM; **P < 0.01, two-tailed unpaired Student’s t-test. [file Image_1.tif]
